# Allopreening in the Black-browed Albatross (*Thalassarche melanophris*): an exploration of patterns and possible functions

**DOI:** 10.1111/ibi.12960

**Published:** 2021-05-04

**Authors:** Natasha Gillies, Tim Guilford, Paulo Catry

**Affiliations:** 1 Department of Zoology, University of Oxford, Oxford, UK; 2 MARE – Marine and Environmental Sciences Centre, ISPA – Instituto Universitário, Rua Jardim do Tabaco 34, Lisbon, 1149-041, Portugal

**Keywords:** display, foraging, negotiation, parental care

## Abstract

The functions of display between breeding pairs of animals have been given little attention outside of sexual selection. Yet evidence suggests that display between partners is in fact most commonly observed following mate choice, and is often just as elaborate. In many bird species, allopreening, when one member of a pair preens the other, is a major component of display both pre- and post-pair formation. Despite this, there has been little investigation into its functions. Explanations that have been put forward tend to focus on its role in feather hygiene, which has limited phylogenetic support, or its function in the maintenance of the pair bond, though how this might occur or indeed what this actually represents has not been adequately explained. Phylogenetic evidence reveals that allopreening is most commonly observed in those species exhibiting high levels of partner retention and biparental care, and it appears to be functional in maintaining cooperation in parental behaviour in at least one species. In our observational study, we explored the patterns and putative functions of allopreening during the nest-relief displays of breeding pairs of Black-browed Albatrosses *Thalassarche melanophris* during incubation and chick-provisioning. Allopreening was an important feature of displays, constituting 30% of display time. We found that the bird returning from its foraging trip usually initiated allopreening, and preened more than its partner prior to change-over of nesting duties. We further found a positive relationship between the amount of time the pair spent in display and the duration of the subsequent foraging trip, providing tentative support for a function in maintaining cooperative parental behaviour between the parents. Although we cannot be conclusive as to its exact functions, we add to a limited literature the first exploration of functions for this conspicuous behaviour in albatrosses.

Despite more than a hundred years having passed since Huxley’s first observations that intra-pair display is more commonly observed after than before pair formation (Huxley 1914), pair display often remains viewed through the lens of sexual selection (Griffith 2019). Consequently, the functions of displays occurring after mate choice has been made (hereby post-pair formation display) remain poorly understood. Post-pair formation displays can be every bit as elaborate as those seen during mate choosing, from the post-nuptial displays of Great Crested Grebes *Podiceps cristatus* that initially piqued Huxley’s interest, to the private duets of Zebra Finches *Taeniopygia guttata* as they exchange incubation duties (Elie *et al*. 2010). Although such displays have historically been explained with reference to the intangible ‘maintenance of the pair bond’ (Harrison 1965), this in itself requires ultimate explanation, and few authors have attempted to explain exactly how display contributes to this maintenance, or indeed what the bond actually represents (Wachtmeister 2001).

In birds, one of the more common forms of post-pair formation display is the nest-relief ceremony (Wachtmeister 2001), in which one parent returns to the nest to relieve the other of its parenting duties. These displays are common in socially monogamous, biparentally caring species. For such species, in which pair bonds persist for long time periods, the breeding success of parents is largely interdependent, ultimately leading to highly aligned lifetime reproductive output (Griffith 2019). Consequently, parents may benefit from coordinating their behaviour to maximize the benefit to cost ratio of providing care from the perspective of both parents (Mariette & Griffith 2015, Pilakouta *et al*. 2018). Intra-pair communication can help parents achieve this coordination and may manifest in post-pair formation display. Through such displays, parents may, for example, signal their state to the partner to stimulate a change in its behaviour. This appears to be the case in Great Tits *Parus major*, where the incubating female vocally communicates her hunger to the male to stimulate him to feed her more, so that she can reduce her own foraging effort (Boucaud *et al*. 2016a). Communication may also facilitate direct negotiation: Blue-footed Boobies *Sula nebouxii* ultimately come to a collaborative decision on where to lay their eggs by negotiating their nesting site preferences through ‘nest-pointing’ displays (Drummond *et al*. 2002).

Allopreening, in which one individual preens another, is often incorporated into displays both pre- and post-mate selection (Wachtmeister 2001). Allopreening may play a hygienic role through the removal of ectoparasites, as has been experimentally investigated in some species (e.g. Rock Pigeons *Columba livea*, Villa *et al*. 2016; *Eudyptid* penguins, Brooke 1985), or may help maintain feather condition by protecting against breakage and facilitating the distribution of preen oil (Clayton 1991). However, its restricted phylogenetic distribution suggests any such function must be secondary to its ancestral purpose (Harrison 1965). Furthermore, if allopreening initially evolved to serve a hygienic function, it might be expected that its occurrence would be particularly prevalent in highly gregarious species living in close physical proximity, as a means to control the spread of ectoparasites. However, phylogenetic analysis (Kenny *et al*. 2017) reveals no association between allopreening and colonial living. Both allopreening and its mammalian counterpart, allogrooming, have been found to reduce stress in a variety of taxa, including Brünnich’s Guillemot *Uria lomvia* (Kober & Gaston 2003), Common Guillemots *Uria aalge* (Lewis *et al*. 2007), Ravens *Corvus corax* (Stöwe *et al*. 2008), Horses *Equus caballus* (Feh & de Mazierès 1993) and Crested Black Macaques *Macaca nigra* (Aureli & Yates 2010). However, it is not clear how or why allopreening would evolve to elicit such a response. Social functions for allogrooming in mammals have been well-explored, particularly in the primates (Dunbar 1991), and increasing evidence is emerging for the importance in birds of its counterpart, for example when forming and maintaining social bonds (Morales Picard *et al*. 2020), establishing dominance (e.g. Large-billed Crows *Corvus macrorynchos*, Miyazawa *et al*. 2020), forming pairs (e.g. Eurasian Wrens *Troglodytes troglodytes*, Gill 2012), and as an appeasement behaviour (e.g. Common Guillemots, Birkhead 1978). However, the possibility that allopreening functions in maintaining cooperation in parental behaviour has been little explored. A study of allopreening in Common Guillemots revealed the first evidence that allopreening may serve a cooperative function (Takahashi *et al*. 2017). Parent Common Guille-mots that return to the nest without food for the chicks spent more time preening the partner, and birds whose partners took longer to take over nesting duties delayed allopreening, suggesting it may serve a dual appeasement and punishment function. Allopreening has additionally been linked to partner retention between breeding seasons (Gill 2012) and high levels of parental cooperation (Kenny *et al*. 2017), further hinting at a social and potentially cooperative function of this behaviour.

Allopreening is a common behaviour in the Black-browed Albatross *Thalassarche melanophris* that can be observed during initial pair formation, between paired individuals throughout the breeding season, and between parents and offspring (Tickell 1984). While allopreening albatrosses tend to focus on the head and nape of their partners, as is the case in other species (Harrison 1965), direct observations conducted by Tickell (1984) did not report any evidence of individuals removing lice or ticks from their partners during allopreening, suggesting this is not its primary function, though this is yet to be experimentally tested. As is characteristic of the Procellariiforms, Albatrosses are long-lived, have stable, long-term pair bonds, and care for their offspring cooperatively with their partner (Tickell 2000). That paired individuals continue to allopreen following their reunion at the beginning of the season and throughout breeding raises the prospect that allopreening may function to encourage or facilitate parental care. Whereas some evidence from other species points to mechanistic explanations for how this could operate, for example by stimulating the production of hormones such as oxytocin or prolactin (Keverne *et al*. 1989), or by reducing stress levels (Schino *et al*. 1988), functional hypotheses have been less well explored, particularly in birds.

During incubation and brood guarding, parent Black-browed Albatrosses engage in conspicuous nest-relief displays as they exchange parenting duties with the partner. While these displays comprise many other behaviours, such as vocalization and ritualized dance postures, the predominant behaviour is allopreening, on which this study is focused. Using a field observation approach, we describe the patterns of allopreening observed between reuniting parent albatrosses during late incubation and early brood guarding and consider the potential drivers and functions of its observed variation.

First, levels of allopreening have been found to vary with sex (Zolnierowicz *et al*. 2016, Miyazawa *et al*. 2020), have been linked to partner retention (Gill 2012, Kenny *et al*. 2017), and may vary with age and breeding experience (Lanctot *et al*. 2000, Perrot *et al*. 2016). We therefore investigated whether these factors predict the duration of allopreening bouts and nest-relief displays in the Black-browed Albatross.

Secondly, based on previous phylogenetic analyses that report that allopreening is associated with cooperative parental care (Kenny *et al*. 2017), we investigated the possibility that allopreening may serve such a function in Black-browed Albatrosses, which, as long-lived, socially monogamous birds, should be selected to care cooperatively with their partners (Griffith 2019). Specifically, we explored whether allopreening might facilitate information transfer that could be involved in the coordination of parental care. For example, allopreening might form part of an assessment or negotiation process whereby the outgoing parent determines the condition or state of its partner, and subsequently uses this information to decide how long to spend at sea. In this way, an outgoing bird that perceives its partner to be in a poor state may spend less time at sea so it can relieve it from nesting duties more quickly. Evidence for such prior ‘planning’ of foraging trip duration has been reported for Manx Shearwaters *Puf*fi*nus puf*fi*nus* (Guilford *et al*. 2008), where parents that embarked on long foraging trips were already found further from the colony than those on short trips on the first day of foraging. To investigate the possibility that the nest-relief ceremony could facilitate information transfer in this way, we investigated whether a relationship existed between display time and the duration of the subsequent or previous forward trip. Furthermore, when the egg hatches, allopreening between adults and chicks could present an additional source of information for such decision-making processes. For example, parents’ willingness to engage in allopreening of chicks might signal to their partner that they are in good condition and therefore ready to provide care. We explored the possibility of a signalling role for chick-directed allopreening by examining whether there were qualitative differences in chick-directed allopreening when it occurred during the nest-relief display vs. when parents were alone with their offspring. Such mechanisms of assessment could allow parents to make optimal decisions about their foraging trip durations from the perspective of the pair as a whole, ultimately maximizing life-time reproductive output for both parents.

## Methods

### Study site

This study was carried out on New Island, Falkland Islands (51°43’32”S, 61°17’55”W), where individually marked Black-browed Albatrosses have been monitored since 2003. Between 6 December 2019 and 10 January 2020, encompassing late incubation and early chick-rearing, which in this species lasts 68–71 and 120–130 days, respectively (Tickell 2000), adult attendance was monitored daily in 114 nests in which both parents were marked with a coloured plastic ring, permitting identification at a distance. The sex of birds had been previously identified through the observation of sex-specific pre-incubation behaviours or through molecular techniques using DNA from blood samples (Fridolfsson & Ellegren 1999). For 74 nests, pairs had been monitored for multiple years and so their pair experience, taken as the number of years both birds had been observed breeding together, could be determined. The age of eggs and chicks was taken as the number of days since laying and hatching, respectively.

### Observations

A total of 99 nest-relief displays were observed. Observations were conducted between the hours of 09:00 and 20:00 (Falkland Islands Standard Time) by a single observer. Approximately 6 h of observation was conducted per day, usually split into two shifts. A mean of 2.8 displays were observed per day. Due to the small size of the colony and its sloped topography, all nests could be observed from a single viewpoint. Behavioural data were collected during the nest-relief displays of pairs, when the previously foraging parent returns to the nest to relieve its partner from parenting duties (incubating the egg or brooding the chick). Data collection began from the moment the incoming bird was sighted near the nest to the moment its partner was deemed to have departed from the colony, either because it had taken off into flight or because it had walked out of sight. The time between the arrival of the incoming bird at the nest and the point at which the departing bird ceased interaction with its partner was recorded as ‘total display time’. Whenever the incoming bird arrived at the colony, the observer commenced data collection while simultaneously changing position to be within 1–2 m of the target nest for the duration of the recording period. At this distance, pairs did not respond to observer movements (N. Gillies pers. obs.). This movement took less than 1 min and observation was possible throughout, so important behaviours were unlikely to be missed. Allopreening was identified as any preening directed at either the partner or chick. For any bout of allopreening, the duration and recipient were manually recorded in real time using a pencil and notepad, and a stopwatch to record duration, for both parents simultaneously. Any switch or pause in behaviour that lasted longer than 1 s indicated the end of a single bout. The time at which parents physically exchanged duties (indicated by swapping positions on the nest) was recorded and is henceforth referred to as the ‘changeover’.

The duration of foraging trips was determined as the number of days between sequential sightings of an individual Black-browed Albatross at the nest, i.e. the total number of consecutive days that that individual was not seen on the nest. Mean foraging trip duration is 5.2 days (range: 0.3–11.3 days) during incubation and 1.9 days (range: 0.1–6.3 days) during chick provisioning (Granadeiro *et al*. 2018), and so while it is possible that adults returned to the nest outside of the observation window, and so the duration of the trip in hours would not be known, number of days is a valid proxy for trip duration (Weimer-skirch *et al*. 1994).

Allopreening between adults and offspring was observed during nest-relief displays and sporadically when parents were alone with the chick during brooding. We investigated whether there were qualitative differences in chick-directed allopreening between these contexts that may indicate a role of this behaviour in the display. To this end, we collected data on the number of chick-directed allopreening bouts during nest-relief displays. We additionally estimated the frequency at which chick-directed allopreening occurs when parents are alone during brooding. Between 1 and 10 January 2020, we scanned the entire colony opportunistically in three to five 5-min bouts per day during the observation window, and counted the number of brood-guarding parents that allopreened their chicks in this time. We converted the resulting number of parents observed allopreening to a proportion of total nests in the colony where the adult was brood guarding. These frequency data were collected over 31 bouts.

### Environmental data

Wind conditions at sea are likely to affect the duration of foraging trips in albatrosses (Wakefield *et al*. 2009), and so we accounted for this in our models of trip duration. Crosswind and tailwind components were reasoned to be the most important components of the wind, based on an *a priori* expectation as to effects of the interaction between wind and a shear-soaring flight mechanism (Pennycuick 2002, Sachs 2004, Paiva *et al*. 2010, Ventura *et al*. 2020). Estimated wind data for the colony location were downloaded from the NOAA Global Forecast System at a spatial resolution of 0.5° and a temporal resolution of 3 h using the R package rWind (Fernández-López & Schliep 2019). For each bird, a probable crosswind and tailwind component was calculated for its at-sea duration assuming travel to the most likely feeding locale around Staten Island (southern Argentina; Catry *et al*. 2013). It was further hypothesized that weather conditions at the colony might affect display behaviours. To this end, qualitative data on weather were collected each morning and afternoon of observations, using the simple categorization of ‘overcast’, ‘sunny’, ‘showers’ or ‘fog’.

### Statistical analysis

Statistical analyses were completed in R version 3.51 (R Core Team 2018). The R package lme4 (Bates *et al*. 2015) was used to construct linear mixed effects models (LMMs) and generalized linear mixed effects models (GLMMs), and *P*-values were obtained by comparing full models containing all variables to null models without the effect of interest, using a likelihood ratio test. For categorical variables, least squares means for each level of the factor were calculated using the R package emmeans (Lenth *et al*. 2018). Data are presented as means ± standard deviation. For brevity, model structures are presented in Table 1. All models were fitted with a random intercept for individual (Ring), pair identity (Pair) or individual nested within pair identity (Pair:ring) as appropriate, to control for repeated measures. We additionally included the fixed effects of sex, age and breeding experience (in years), which we identified as factors possibly influencing behaviour.

We investigated which variables explain the amount of time spent allopreening by individuals (Table 1, Model 1), by the pair as a whole (Table 1, Model 2), and between parents and off-spring (Table 1, Model 3).

To investigate whether sex or the breeding experience (in years) of individuals influenced the amount of time they engaged in allopreening, we fitted a binomial GLMM to the time an individual spent allopreening its partner, calculated as a proportion of the total allopreening time in the display, as a function of sex and breeding experience (Table 1, Model 1). Allopreening time may differ depending on whether the bird is arriving at or departing from the nest and might additionally vary before and after physical changeover on the nest. We accounted for this by including the fixed effects of ‘position’ (incoming vs. outgoing bird) and ‘timing’ (pre- or post-changeover).

‘Total allopreening time’ was taken as the summed duration (in seconds) of all allopreening bouts by both parents in a single display. We investigated whether total allopreening time varied with breeding experience of the pair (in years), historical breeding success (chicks fledged per breeding attempt) or sex of the outgoing bird by fitting an LMM (Table 1, Model 2) that included these variables as fixed effects. To account for differences between incubation and chick provisioning, we additionally included ‘breeding stage’ as a fixed effect. Finally, we included total display time (in minutes) as a controlling variable, as this dictates the amount of time available for allopreening.

We investigated whether and which factors predicted the number of chick-directed allopreening bouts instigated by each parent. We fitted an LMM to the number of bouts using the predictors of sex and whether the bird was arriving at or departing from the nest (‘position’; Table 1, Model 3). We included ‘total display time’ as a fixed effect to account for the amount of time available to parents to allopreen their chicks.

We were further interested in what factors predict variation in total display time (Table 1, Model 4) and foraging trip duration (Table 1, Model 5). We hypothesized that if nest-relief displays facilitate information transfer between the pair, then we would observe a relationship between the duration of the display and the duration of either the preceding or the subsequent foraging trip.

For models 4 and 5, we included only those nests for which previous trip duration was known. As only 16 nests had more than one observation, we randomly selected one observation from each of these nests. We first fitted a linear model (LM) to total display time (minutes) as a function of the duration of the previous foraging trip in days (‘previous trip’; Table 1, Model 4). As display time may also vary with breeding experience of the pair, historical breeding success, breeding stage, offspring age and sex of the outgoing bird, we included these variables as fixed effects. The effect of age may differ between eggs and chicks, and so we included an interactive effect between age and breeding stage. We then fitted an LM to subsequent foraging trip duration (days) as a function of total display time (minutes) and total allopreening time (seconds; Table 1, Model 5). As before, we controlled for experience, breeding stage, historical breeding success, offspring age and sex of the outgoing bird. As consecutive foraging trips are likely to be correlated in length, we also included the fixed effect of previous trip duration. Finally, environmental conditions are known to affect foraging trip duration in Black-browed Albatrosses (Wakefield *et al*. 2009). To control for this, we included the environmental variables of crosswind, headwind and qualitative weather (see Methods, Environmental data).

## Results

Displays in which the exact timings of changeover were not known or where foraging trip duration was unknown were excluded from the analyses, leaving 91 displays across 63 nests. Across all displays, the incoming bird was female in 49% of observations and male in 51% of observations.

Of the 91 nest reliefs observed, 90 involved display, which lasted for an average of 23.3 ± 19.0 min (range: 3–101 min). In the single nest relief that did not involve display, the sitting bird left the nest immediately following its partner’s arrival at the colony. Allopreening was observed in all 90 of these displays and constituted a mean of 26.7 ± 16.2% of display time (range 0.26–79.33%). Anecdotally, it was observed that this tended to focus on the head or neck of the bird, but it was not clear that any birds attempted to target ectoparasites specifically; parasites visible by an observer at a distance of 1–2 m were on occasion seemingly ignored by the allopreening bird (N. Gillies pers. obs.). Individual bouts of allopreening lasted 16.1 ± 23.4 s, and each display comprised 27.1 ± 26.2 bouts.

In 62% of displays, the incoming bird initiated allopreening, in 26% the outgoing bird initiated, and in 12% both birds began allopreening simultaneously. Following the changeover, the incoming bird resumed allopreening first in 64% of displays, the outgoing bird resumed in 28% of displays, and both birds simultaneously resumed in 8% of displays.

### Model 1 – Individual allopreening time

**Figure 1:**
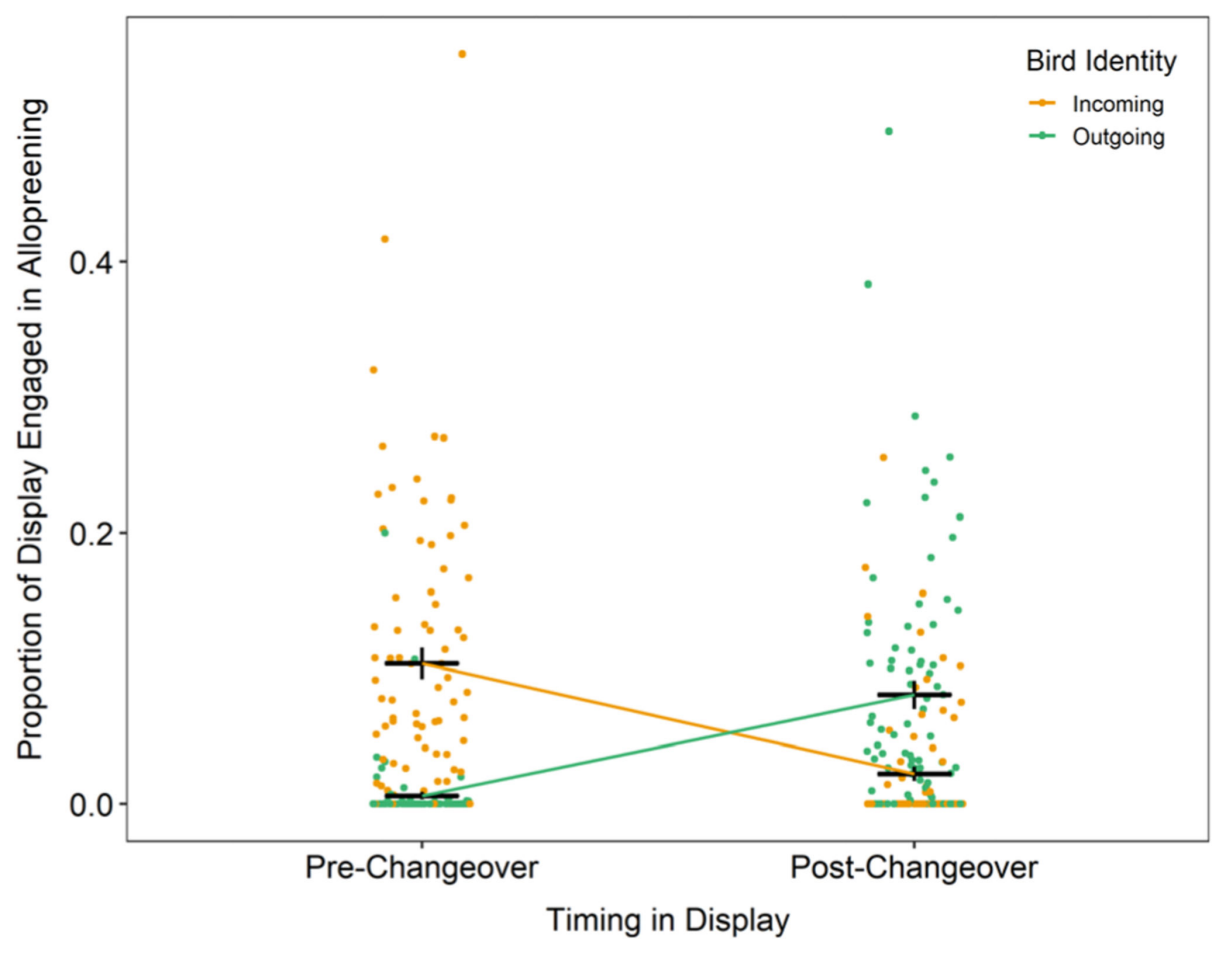
Proportion of total display time spent allopreening prior to changeover and following it for the incoming (orange) and outgoing (green) parents. Black crosses indicate mean (horizontal line) and standard error (vertical line). Data points are ‘jittered’ horizontally for readability.

The proportion of time individuals spent allopreening their partners during the display was best predicted by an interaction of whether the bird was incoming to or outgoing from the nest, and whether allopreening occurred before or after changeover (χ^2^ = 12475.05, df = 1, *P* < 0.0001; Fig. 1). Prior to changeover, incoming birds spent a greater percentage of the display allopreening compared with departing birds (incoming: 5.27 ± 0.84%, outgoing: 0.24 ± 0.042%). This reversed following changeover, when outgoing birds were found to spend more time allopreening compared with incoming birds (incoming: 2.41 ± 0.39%, outgoing: 4.44 ± 0.72%). Females spent a greater percentage of their display time engaged in allopreening compared with males (female: 2.97 ± 0.63%, male: 1.26 ± 0.28%; χ^2^ = 8.3, df = 1, *P* = 0.0040). The number of years an individual had been observed breeding had no effect on the time they spent allopreening (χ^2^ = 0.92, df = 1, *P* = 0.34), and allopreening times were not repeatable at the level of individual (104 observations across 44 individuals, *r* = 0.0087 ± 0.019, *P* = 0.41).

### Model 2 – Total allopreening time

**Figure 2:**
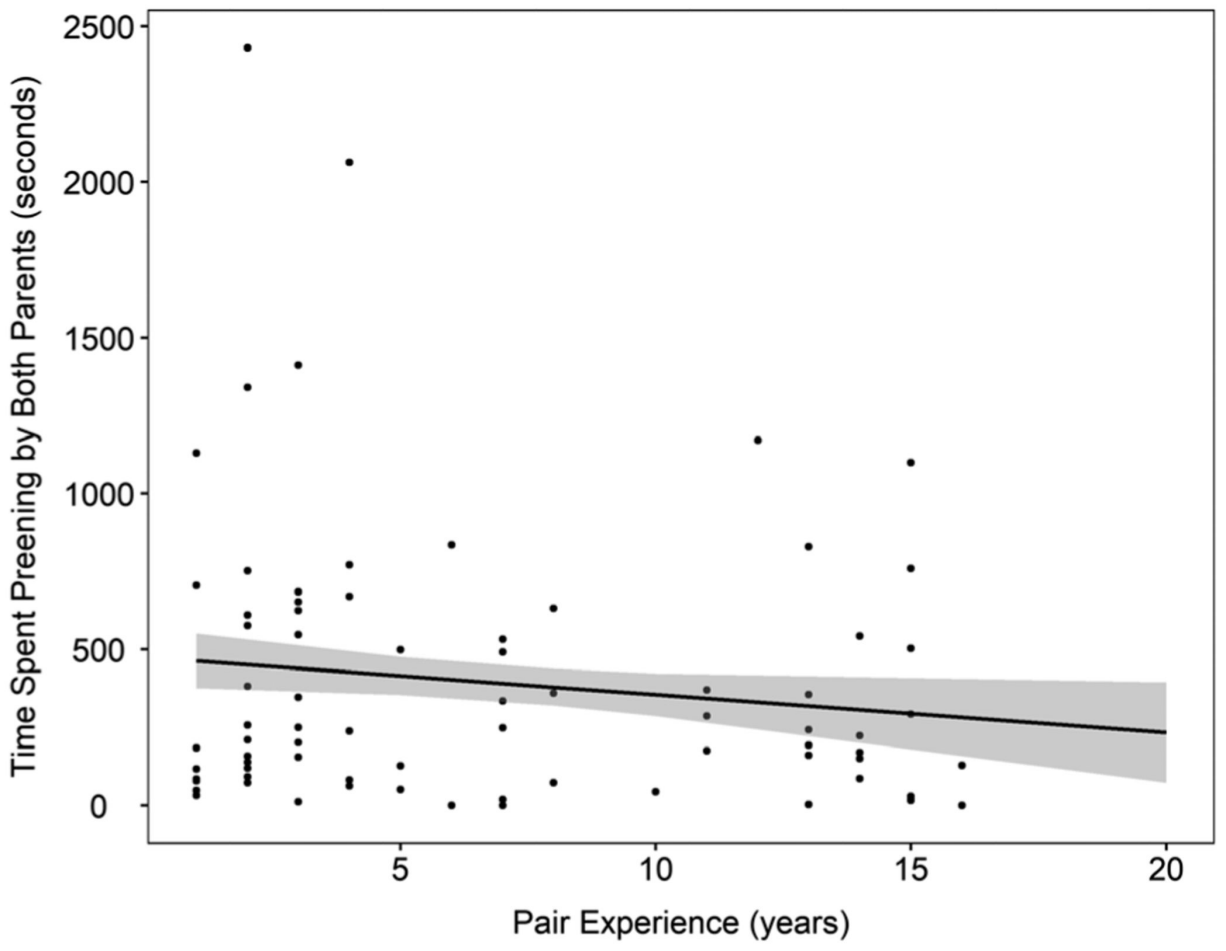
Total time spent preening by both parents in seconds according to pair breeding experience (years). Shaded area gives confidence intervals.

The amount of time pairs spent allopreening decreased by 12.13 ± 5.73 s for each year that the pair had been together, though there was no effect of historical breeding success (Fig. 2; Table 2). There was no effect of the sex of the outgoing bird or breeding stage on allopreening time (Table 2). As expected, there was a strong correlation between total allopreening time and total display time, with the amount of time spent preening increasing by 0.30 ± 0.032 s for each 1-s increase in display time (Table 2), suggesting that when controlling for other variables, the percentage of time spent allopreening during display was approximately 30%.

### Model 3 – Chick-directed preening

Of the 68 displays that took place during chick-rearing, 83.3% involved some allopreening of the chick. The amount of allopreening varied significantly depending on whether it was provided by the incoming, outgoing or both parents (Table 3), with incoming birds providing more bouts of allopreening compared with outgoing parents (incoming: 5.68 ± 0.37 bouts, outgoing: 0.35 ± 0.37 bouts) and simultaneous preening by both parents making up the fewest number of bouts (0.32 ± 0.37 bouts). There was no effect of sex, age, breeding experience or total display time on the number of bouts (Table 5).

Parents were frequently observed to preen chicks when alone during brood guarding. During our daily preening observations, the number of nests in which the chick was being brood-guarded varied between 19 and 79, with a median of 43.

On each day in any 5-min interval, a median of 14.1% of adults could be observed to preen their offspring (range = 8.5–18.4%).

### Model 4 – Display duration

There was no effect of sex of the outgoing bird, previous foraging trip duration, pair experience, or the interaction of breeding stage and offspring age on display duration (Table 4). Displays during incubation were significantly longer than during chick-rearing, at 59.61 ± 20.8 min vs. 15.98 ± 2.69 min for chick-rearing.

### Model 5 – Foraging trip duration

**Figure 3:**
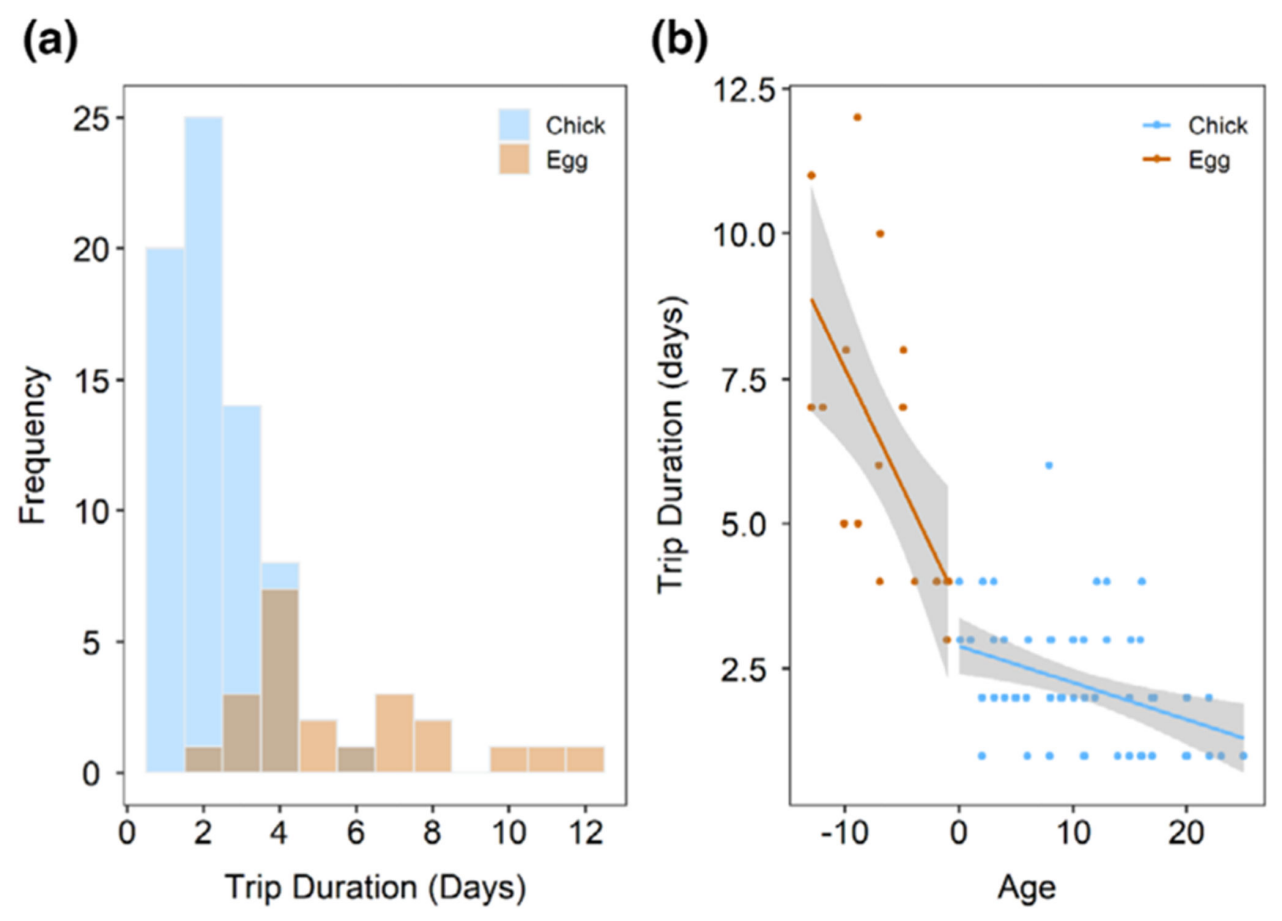
(a) Frequency of foraging trip durations for incubating (orange) and chick-brooding (blue) birds. Colours are transparent to make all bars visible; darker colours indicate overlapping bars. (b) Relationship between foraging trip duration (days) and offspring age (days) for incubating (orange) and chick-brooding (blue) birds. Shaded areas give confidence intervals.

**Figure 4:**
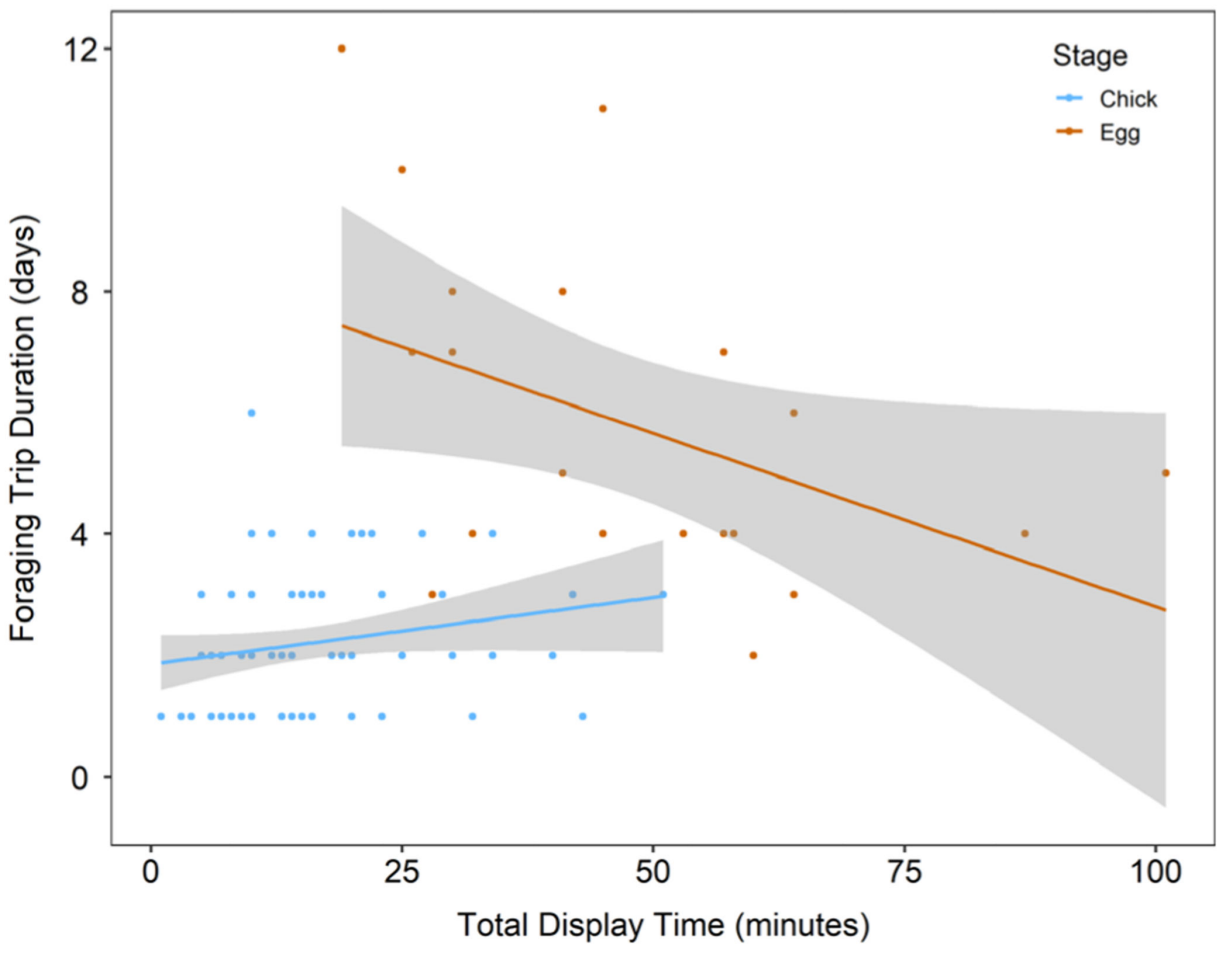
Relationship between total display time (minutes) and foraging trip duration (days) for incubating (orange) and chick-brooding (blue) birds. Shaded area gives confidence intervals.

There was a significant effect of the interaction between offspring age and breeding stage on the duration of foraging trips (Table 5). Mean trip duration for incubating birds was 4.17 ± 1.89 days vs. 1.68 ± 0.39 days for brood-guarding birds (Fig. 3a). For each day increase in age, trip duration decreased by 0.58 ± 0.14 days for incubating birds and increased by 0.0016 ± 0.030 days for brood-guarding birds (Fig. 3b). Total display time significantly predicted foraging trip duration during incubation (Fig. 4, Table 5), with trip duration decreasing by 0.085 ± 0.025 days for each 1-min increase in display time for incubating birds. There was no effect of sex, pair experience, previous trip duration, total allopreening time or any of the three weather variables on trip duration (Table 5).

## Discussion

We report tentative evidence that allopreening, which was observed to be a conspicuous feature of nest-relief displays in Black-browed Albatrosses, may facilitate parental cooperation over care. For incubating birds, the amount of time spent in display was negatively correlated with the duration of the subsequent foraging trip, but was not related to the duration of the foraging trip preceding the changeover. This relationship might indicate that this behaviour facilitates information transfer that may relate to subsequent decisions about foraging trip duration. Both parents directed bouts of allopreening of one-another, sometimes simultaneously, for variable amounts of time. The parent returning from its foraging trip tended to initiate allopreening both during the display and following the switching of parents (‘changeover’) on the nest. Furthermore, this incoming bird allopreened its partner the most overall, though there was a divide between the pre- and post-changeover periods: following changeover, the outgoing bird allopreened relatively more than its now-sitting partner. Most allopreening focused on the head and neck of the partner, areas that are inaccessible during self-directed preening (autopreening). However, this did not seem to be specifically targeted at ectoparasites, matching previous observational accounts of allopreening in Black-browed Albatrosses (Tickell 1984).

As albatrosses are long-lived and highly socially monogamous (Bried *et al*. 2003, Burg & Croxall 2006), pairs are expected to share a large proportion of their reproductive output over their breeding lifespan (Griffith 2019). Consequently, the benefit to either bird of exploiting its partner by reducing its own care, for example by taking an excessively long foraging trip, is limited, as any costs imposed on the partner are likely to be subsequently shared with the foraging bird. Such costs may be incurred either when the partner takes its own long foraging trip, which *in extremis* could lead to an increased risk of desertion (Weimer-skirch 1995), or in future breeding attempts, if the partner’s condition is reduced substantially. As such, conflict between the parents is reduced, and so optimal foraging trip duration is likely to reflect a compromise between the needs of both parents, as has been previously reported for Antarctic Petrels *Thalassoica antarctica* (Tveraa *et al*. 1997). However, establishing such a compromise requires exchange of information between the two partners.

Recent evidence suggests that nest-relief displays may facilitate such a process in other species. In Common Guillemots, long nest-relief ceremonies, which are largely made up of bouts of allopreening, are associated with poor body condition of the incoming bird (Takahashi *et al*. 2017). Conversely, Zebra Finches that are delayed in their return to the nest engage in shorter changeover duets with the partner and shorter subsequent foraging trips (Boucaud *et al*. 2016b). Nest-relief displays may serve a similar function in Black-browed Albatrosses, allowing the outgoing parent to decide on its optimal foraging trip duration by assessing the state of its partner. That most allopreening observed was delivered by the incoming bird may suggest that behavioural information, such as the incoming partner’s ability or willingness to allopreen, is key to assessing partner state, as a physical assessment would probably be reflected in more preening by the outgoing bird. Furthermore, it is unclear how allopreening directed at the head or neck would facilitate physical assessment of condition; if this were the function, then it might be more logical for the outgoing bird to focus on the body of its partner, which might allow assessment of, for example, fat reserves. Instead, allopreening by the incoming bird may be explained as a mechanism of ‘reassurance’, whereby the arriving bird signals to its partner a strong confirmation that it is motivated and able to take over incubation duties, reducing the risk of egg abandonment and therefore nest failure. Through this, the outgoing parent can make an assessment of its partner’s commitment to engage in parental care.

This could additionally provide an explanation for the fact that, following hatching, allopreening of the chicks by either or both parents became a common component of the nest-relief display. Bouts of chick-directed allopreening were most commonly observed by incoming birds, which were reuniting with the chick (and partner). An assessment function for allopreening might explain this behaviour, which could in theory provide information to displaying parents, for example by indicating willingness of the allopreening bird to engage in parental care. However, while the tendency of incoming birds to preen the chick the most during displays supports this, that parents continued to allopreen when alone with the chick means the evidence that this plays a role in display is limited. Indeed, for brood-guarding birds, the effect size of the relationship between foraging trip duration and allopreening time was slightly positive, but small and not statistically significant. Foraging trip durations were considerably shorter during chick-rearing, a common strategy observed in seabirds that can help to balance the costs of commuting and the risks of chick starvation when delivering food (Cuthill & Kacelnik 1990). This may consequently mean less time is available to the parents for display and, by extension, allopreening. Brood-guarding albatrosses experience higher energetic expenditure than incubating adults (Bevan *et al*. 1995), and so despite their shorter spells on the nest they are probably subject to similar constraints as during incubation. Furthermore, if the returning adult arrives with little food available for the chick, it is important that its partner does not subsequently spend long at sea. As such, trip duration may be more constrained due to the competing demands of self-maintenance and chick-provisioning, meaning that both ‘reassurance’ of a commitment to caring by the partner and assessment of partner condition are probably less important. Indeed, displays were considerably shorter during chick-rearing than during incubation, suggesting limited value of an information transfer function, if it exists.

An assessment function for allopreening during incubation may explain the observed inverse relationship between display duration and the length of the subsequent foraging trip. Long-lived species are expected to prioritize future survival and reproduction (Stearns 1992) and so outgoing parents that are in poor condition would be expected to make decisions that favour their own condition, even at the expense of the current breeding attempt. Consequently, such poor condition parents might have less to gain from assessing the condition of their partner and will spend less time displaying so they can leave to forage more quickly. These birds will subsequently spend more time at sea while they replenish their lost mass reserves. Conversely, sitting birds that are in good condition are less constrained by this resource trade-off and so might be less motivated to leave the nest without strong persuasion from the partner that it is ready to take over caring duties. This might manifest in a longer period of display as the incoming bird attempts to convince its partner to exchange places on the nest, followed by a shorter foraging trip as the departing parent does not need to spend as much time at sea to regain mass. Indeed, incubating, and to a lesser extent brooding, adults were often observed to be extremely reluctant to leave the nest, with changeovers occasionally being initiated when the incoming bird physically pushed its partner off the nest (in our study, such behaviour was observed in 11 of 99 displays). Data on adult body condition were not available in our study, but support for a condition-dependent ‘persuasive’ function for allopreening may be seen in the finding that females tended to allopreen more than males. Males are larger than females (Ferrer *et al*. 2016) and probably in better overall condition as they have not borne the cost of producing the egg (Astheimer *et al*. 1985). Consequently, they are more likely to be capable of sustaining incubation for longer, which could manifest in greater reluctance to leave the nest. Males and females did not differ in their foraging trip durations and so would be likely to experience differential costs of incubating that could manifest in such differences in motivation.

Besides behaviour, outgoing birds may make an assessment of their partner using their well-developed olfactory sense, which is an important part of the sensory system of seabirds (Hagelin *et al*. 2003, Nevitt 2008). Outgoing birds may use olfactory cues gathered from the head and neck of their partners to detect cues that may indicate foraging success, such as the type or quality of food, and therefore its probable condition. One hypothesis for the functional significance of signal repetition is that it serves to reduce error in the assessment of the receiver (Enquist & Leimar 1983, Mowles & Ord 2012). It is possible that the inverse correlation between trip duration and total display time observed here reflects the outgoing bird taking longer to assess the partner when it is in poor condition, and therefore soliciting further allopreening from the partner. If the outgoing bird determines that the partner is in a worse state than normal, then this may encourage it to spend less time at sea and thus to return and relieve its partner earlier, reducing the risk of egg abandonment, which condemns the breeding attempt to failure due to predation in this species (Warham 1990, N. Gillies pers. obs.). However, it is probable that if allopreening functioned in this way, more allopreening would be observed by the outgoing bird. Further research is needed to determine whether behavioural or olfactory cues actually relate to body condition, which would provide the necessary evidence that such a role for allopreening could exist.

For parents visiting lone chicks, several minutes of chick-directed allopreening were observed before the parent either began brooding or commenced feeding. This may suggest chick allopreening has an alternative role in reunification, evidence for which has been observed in primates, where temporary separation of mothers and offspring leads to a subsequent increase in grooming behaviour following reunion in a wide range of species (Anderson & Chamove 1979, Gunnar *et al*. 1981, Taylor *et al*. 2015). However, the function of this sort of behaviour is not clear, and furthermore, brood-guarding parents regularly preened their chicks sporadically during the day, with no obvious initiating cue. The functions of offspring-directed allopreening in birds have not been well explored. Possible functions include assessing chick need to ensure optimal food delivery (Weimer-skirch *et al*. 1997), reducing tick load (Bergström *et al*. 1999), though the evidence for this in Black-browed Albatrosses is limited (Tickell 1984; P. Catry pers. comm.) or stress reduction (Taylor *et al*. 2015). Future work should examine how patterns of offspring-directed allopreening change as the chick ages, whether allopreening reduces measures of stress, and whether it correlates with reduced tick load.

Finally, the total amount of allopreening observed during the nest-relief display decreased with each year that a pair had been observed breeding together. In many bird species, reproductive success is seen to increase with duration of the pair bond, even when controlling for possible confounding variables such as age (Emslie *et al*. 1992, Pyle *et al*. 2001, van de Pol *et al*. 2006). However, the mechanistic basis of this is not well known. Earlier laying (van de Pol *et al*. 2006), reduced time spent courting (Sanchez-Macouzet *et al*. 2014) and improved behavioural coordination (Griggio & Hoi 2011) of more experienced pairs have all been used to explain this improvement in breeding success over successive years of mating. It is possible that the reduced amount of preening observed in more experienced pairs reflects an improvement in the coordination of care. For example, birds may take less time to assess the condition of their partners with increased experience, or require less stimulation to leave the nest. However, the effect observed here was small. If there is indeed no relationship, this would suggest that ‘maintenance of the pair bond’, which would be expected to precipitate some relationship between pair duration and allopreening, would not be a satisfactory explanation for the function of this behaviour. Further research is necessary to determine whether the observed relationship is real and, if so, what is its driver. Alternatively, a relationship may exist between allopreening and the amount of time parents remain together in the future. To this end, future studies could consider whether pairs that allopreen more are less likely to divorce in subsequent years.

Our observations provide tentative evidence that intra-pair displays between parent Black-browed Albatrosses, in which allopreening behaviour is a key component, may facilitate cooperation over parental care. Allopreening by incoming parents may be a mechanism by which arriving birds try to ‘convince’ their partner to allow them to take over parenting duties, or alternatively might allow the departing parent to assess its partner’s condition or success at sea, so it can adjust its foraging trip duration accordingly. To determine which, if either, hypothesis is more likely, future work should investigate how body condition of the parents relates to the amount and patterns of allo-preening observed. The phylogenetic distribution of allopreening, as well as previous observations of Black-browed Albatrosses (Tickell 1984), do not support an ancestral role in plumage maintenance, and so we do not suspect this to be its primary role. However, we did not investigate this directly and so further experimental work would be needed to draw conclusions on the putative hygienic or feather maintenance benefits of this behaviour. The function of other facets of the nest-relief display not investigated here such as vocalization and dance postures have not been explored. These aspects of display may supplement the reassurance or assessment functions of allopreening suggested here, or may serve altogether different purposes. Many aspects of the nest-relief display incorporate elements seen also in displays between newly forming pairs. Ultimately, allopreening is likely to be multi-functional; however, our findings suggest for the first time that in this species, it may serve a cooperative function.

## Figures and Tables

**Table 1:** Model structures used in the analysis. Model numbers are those in the text.

Type of model	Model	Parameters		
		Response	Fixed	Random
Binomial GLMM	1	Individual proportion of time spent allopreening	Position * timing + sex + breeding years	Pair : ring
LMM	2	Total allopreening time (in seconds (s))	Experience + success + stage + sex + total display time	Pair
	3	Number of chick-directed preening bouts	Experience + position + age + sex + total display time	Pair : obs.ID
LM	4	Total display time (min)	Experience + success + stage * age + sex + previous trip	
	5	Foraging trip duration (days)	Experience + success + stage * age + sex + total display time + total allopreen time + previous trip + stage * total display time + crosswind + headwind + qual	

Position = whether the bird was ‘incoming’ or ‘outgoing’ from the nest; timing = timing in the display, either pre- or post-changeover; sex = male or female; breeding years = years individual observed breeding; experience = years pair observed breeding together; pair = pair identity; ring = bird identity; success = historical breeding success, number of chicks fledged per breeding attempt; stage = breeding stage, incubation vs chick brooding; total display time = time from arrival of incoming bird to cessation of pair interaction; age = age of offspring relative to hatch date in days; obs.ID = unique identity for each changeover display; previous trip = duration of foraging trip that ended in changeover; total allopreen time = total time spent preening during display; qual = qualitative weather. *Foraging trip duration* refers to the trip immediately following the nest-relief display.

**Table 2:** Test statistics and coefficient coordinates from a likelihood ratio test on LMM estimating predictors of total allopreening time (seconds).

Variable	χ^2^	df	*P*-value	Estimate	se
**Experience**	**4.76**	**1**	**0.029**	–**12.13**	**5.73**
success	0.53	1	0.47	–90.29	134.00
stage (egg)	1.57	1	0.21	104.70	87.85
outgoing sex (M)	3.38	1	0.066	–104.96	57.89
**Total display time**	**59.75**	**1**	< **0.0001**	**18.13**	**2.00**

Significant variables in bold. Experience = number of years pair observed together; Success = historical breeding success, chicks fledged per breeding attempt; Stage = breeding stage, incubation vs. brood guard; outgoing Sex = sex of departing parent; Total display time = total duration of display in minutes.

**Table 3:** Test statistics and coefficient coordinates from a like-lihood ratio test to investigate the significance of fixed effects on the number of chick-directed allopreening bouts.

Variable	χ^2^	df	*P*-value	Estimate	se
Experience	1.23	1	0.26	–0.052	0.048
**Position (incoming)**	**106.49**	**1**	< **0.0001**	**5.36**	**0.49**
**Position (outgoing)**				**0.033**	**0.49**
Sex (M)	0.22	1	0.64	–0.18	0.40
Age	3.61	1	0.057	0.070	0.037
Total display time	0.43	1	0.51	0.015	0.023

Significant variables in bold. Experience = years pair observed breeding together; Position = driver of chick allopreening, either incoming, outgoing, or both parents; Sex = male or female, for simultaneous preening represents sex of incoming bird; Age = offspring age; Total display time = total duration of display in minutes.

**Table 4:** Test statistics and coefficient coordinates from ANOVA to investigate the significance of fixed effects on total display time.

Variable	*t*	P-value	Estimate	se
Experience	0.057	1.0	0.027	0.47
Success	0.92	0.37	9.39	10.27
**Stage (egg)**	**3.38**	**0.0016**	**36.16**	**10.71**
Age	–0.50	0.62	–0.19	0.38
Stage*age	0.61	0.55	0.92	1.52
Outgoing sex (M)	1.88	0.067	8.82	4.69
Previous trip	–0.71	0.48	–0.89	1.25

Significant variables in bold. Experience = years pair observed breeding together; Success = historical breeding success, chicks fledged per breeding attempt; Stage = breeding stage, incubation vs. brood guard; Age = offspring age; outgoing Sex = sex of departing parent; Previous trip = duration of preceding foraging trip.

**Table 5:** Test statistics and coefficient coordinates from ANOVA to investigate the significance of fixed effects on foraging trip duration (days).

Variable	*t*	*P*-value	Estimate	se
Experience	0.72	0.48	0.031	0.043
Success	0.86	0.40	0.74	0.86
**Stage (egg)**	**4.06**	**0.00040**	**4.46**	**1.10**
Age	0.045	0.96	0.0016	0.035
Stage*age	–**4.24**	**0.00025**	–**0.58**	**0.14**
Outgoing sex (M)	1.14	0.27	0.49	0.43
Total allopreen time	1.18	0.25	0.00091	0.00077
Previous trip	1.78	0.086	0.21	0.12
Total display time	–0.32	0.75	–0.0087	0.027
Stage*total display time	–**3.0**	**0.0061**	–**0.076**	**0.025**
Headwind	1.79	0.087	0.0034	0.0019
Crosswind	0.63	0.54	0.00082	0.0013
Qual (showers)	–2.0	0.060	–1.6	0.81
Qual (sunny)	–1.41	0.17	–0.79	0.56

Significant variables in bold. Experience = number of years pair observed together; Success = historical breeding success, chicks fledged per breeding attempt; Stage = breeding stage, incubation vs. brood guard; Age = offspring age; Outgoing sex = sex of departing parent; Total allopreen time = total time spent preening during display; Previous trip = duration of preceding foraging trip; Total display time = time from arrival of incoming bird to cessation of pair interaction; qual = qualitative weather.

## Data Availability

The data that support the findings of this study are available from the corresponding author upon reasonable request.
